# *CDH2* and *CDH13* as potential prognostic and therapeutic targets for adrenocortical carcinoma

**DOI:** 10.1080/15384047.2024.2428469

**Published:** 2024-11-15

**Authors:** Yongli Situ, Li Deng, Ziqing Huang, Xiaoli Jiang, Liubing Zhao, Juying Zhang, Lingling Lu, Quanyan Liang, Qinying Xu, Zheng Shao, Meng Liang

**Affiliations:** a Department of Parasitology, Guangdong Medical University, Zhanjiang, China; b School of Medical Technology, Guangdong Medical University, Dongguan, China

**Keywords:** Bioinformatics, cadherin, cancer, cell cycle, gene regulatory network, immunotherapy, prognosis, transcription

## Abstract

Cadherin 2 (CDH2, N-cadherin) and cadherin 13 (CDH13, T-cadherin, H-cadherin) affect the progress and prognoses of many cancers. However, their roles in adrenocortical carcinoma (ACC), a rare endocrine cancer, remain unclear. To decipher the roles of these proteins in ACC and to identify their regulatory targets, we analyzed their expression levels, gene regulatory networks, prognostic value, and targets in ACC, using various bioinformatic analyses. *CDH2* was strongly downregulated and *CDH13* was strongly upregulated in patients with ACC; the expression levels of these genes affected the prognosis. In 75 patients, the expression of *CDH2* and *CDH13* was altered by 8% and 5%, respectively. *CDH2* and *CDH13*, as well as their neighboring genes, were predicted to form a complex network of interactions, mainly through coexpression and physical and genetic interactions. *CDH2* and its altered neighboring genes (ANGs) mainly affect tumor-related gene expression, cell cycle, and energy metabolism. The regulation of tumor-related integrin function, gene transcription, metabolism, and amide and phospholipid metabolism are the main functions of *CDH13* and its ANGs. MiRNA and kinase targets of *CDH2* and *CDH13* in ACC were identified. *CDH13* expression in patients with ACC was positively associated with immune cell infiltration. Anti-PD1/CTLA-4/PD-L1 immunotherapy significantly downregulated the expression of *CDH13* in patients with ACC. Foretinib and elesclomol were predicted to exert strong inhibitory effects on SW13 cells by inhibiting the expression of *CDH2* and *CDH13*. These data indicate that CDH2 and CDH13 are promising targets for precise treatment of ACC and may serve as new biomarkers for ACC prognosis.

## Introduction

Adrenocortical carcinoma (ACC) is a rare endocrine tumor with a global incidence of 0.7–2.0 cases/million/year.^1^ Approximately 60% of ACC cases are functional. There is a wide range of clinical syndromes depending on the type of hormones produced.^2^ The prognosis of patients with ACC is poor, with a 5-year survival rate of < 40%.^3^ For most patients, there is no effective treatment to prolong survival, and complete surgical resection is the only treatment option.^4^ Therefore, it is necessary to determine the mechanisms underlying the occurrence and development of ACC, and to identify therapeutic targets.

Cadherin is a tumor suppressor that regulates tissue development and differentiation. Currently, more than 100 cadherins are identified, which are categorized into four groups, namely classical cadherins, protocadherins, desmosomal cadherins, and cadherin-related proteins.^5^ Increasing evidence suggests that an imbalance in cadherin expression caused by gene alterations can lead to tumor growth, invasion, and metastasis.^6,7^ Cadherin 2 (CDH2, N-cadherin) is a member of the classical cadherin group that maintains the integrity of cells and participates in many signal transduction pathways. Abnormal expression of *CDH2* has been reported in many cancers, including that of the lung, breast, and prostate, as well as squamous cell carcinoma.^7^ Abnormal expression of *CDH2* can regulate the progression of malignant tumors by affecting apoptosis, angiogenesis, invasion, and metastasis of tumor cells.^8^ Therefore, *CDH2* may be used as a therapeutic target and prognostic biomarker for multiple tumors.^9^ Cadherin 13 (CDH13, T-cadherin, H-cadherin) is a new member of the cadherin superfamily that maintains normal tissue structure. Abnormalities in *CDH13* have been observed in many types of human malignant tumors.^10^ Recently, *CDH13* has been shown to play a role as an anticancer gene in lung, breast, ovary, bladder, and gastric cancer.^11,12^ Abnormal expression of *CDH13* plays a key role in cancer development by promoting the inactivation of tumor suppressor genes, activation of oncogenes, and increasing chromosome instability.^13^

The roles of *CDH2* and *CDH13* in ACC are not well understood. Therefore, in this study, we systematically analyzed the expression, gene regulatory network, prognostic value, putative targets, and potential therapeutic agents of *CDH2* and *CDH13* in patients with ACC. Moreover, we examined the association of ACC with *CDH2* and *CDH13* and identified potential new targets and drugs for ACC therapy.

## Results

### CDH2 and CDH13 expression, prognosis, and genetic alterations in ACC

**Figure 1.**
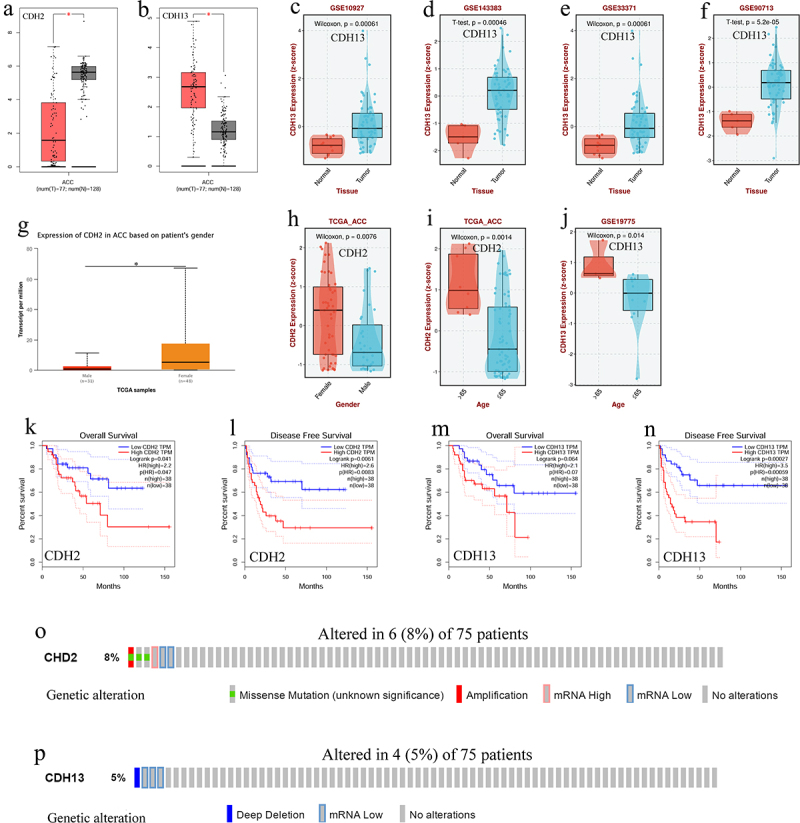
The transcription levels, prognostic value, and genetic alteration of *CDH2* and *CDH13* in adrenocortical carcinoma (ACC). (a) Boxplot showing transcription level of *CDH2* in patients with ACC (GEPIA); (b) Boxplot showing transcription level of *CDH13* in patients with ACC (GEPIA); (c – f) Boxplot showing transcription level of *CDH13* in patients with ACC (BEST); (g and h) Boxplot showing transcription level of *CDH2* in patients with ACC based on sex (UALCAN and BEST); (i) Boxplot showing transcription level of *CDH2* in patients with ACC based on age (BEST); (j) boxplot showing transcription level of *CDH13* in patients with ACC based on age (BEST); (k) the overall survival curve of *CDH2* in patients with ACC (GEPIA); (l) the disease-free survival curve of *CDH2* in patients with ACC (GEPIA); (m) the overall survival curve of *CDH13* in patients with ACC (GEPIA); (n) the disease-free survival curve of *CDH13* in patients with ACC (GEPIA); (o) Genetic alteration of *CDH2* in patients with ACC (cBioportal); (p) Genetic alteration of *CDH13* in patients with ACC (cBioportal); *p < .05.

The transcript level of *CDH2* was significantly downregulated (*p* < .05; Figure 1a) and that of *CDH13* was significantly upregulated (*p* < .05; Figure 1a–f) in patients with ACC. *CDH2* transcript levels were significantly lower in males than in females (*p* < .05; Figure 1g,h). The transcript levels of both genes were significantly downregulated in older patients (≥65 years) compared with those in younger patients (<65 years) (*p* < .05; Figure 1i,j). Furthermore, the overall survival was longer for patients with ACC exhibiting low *CDH2* expression than for those with high expression (*p* = .041; Figure 1k). Disease-free survival was longer in patients with ACC having low *CDH2* and *CDH13* expression than in those with high expression of these genes (*p* = .0061 and *p* = .00027, respectively; Figures 1l–n). Moreover, *CDH2* and *CDH13* expression was altered by 8% and 5%, respectively, in patients with ACC (Figure 1o,p).

### Interaction network of CDH2 and CDH13 and their altered neighboring genes in ACC

**Figure 2.**
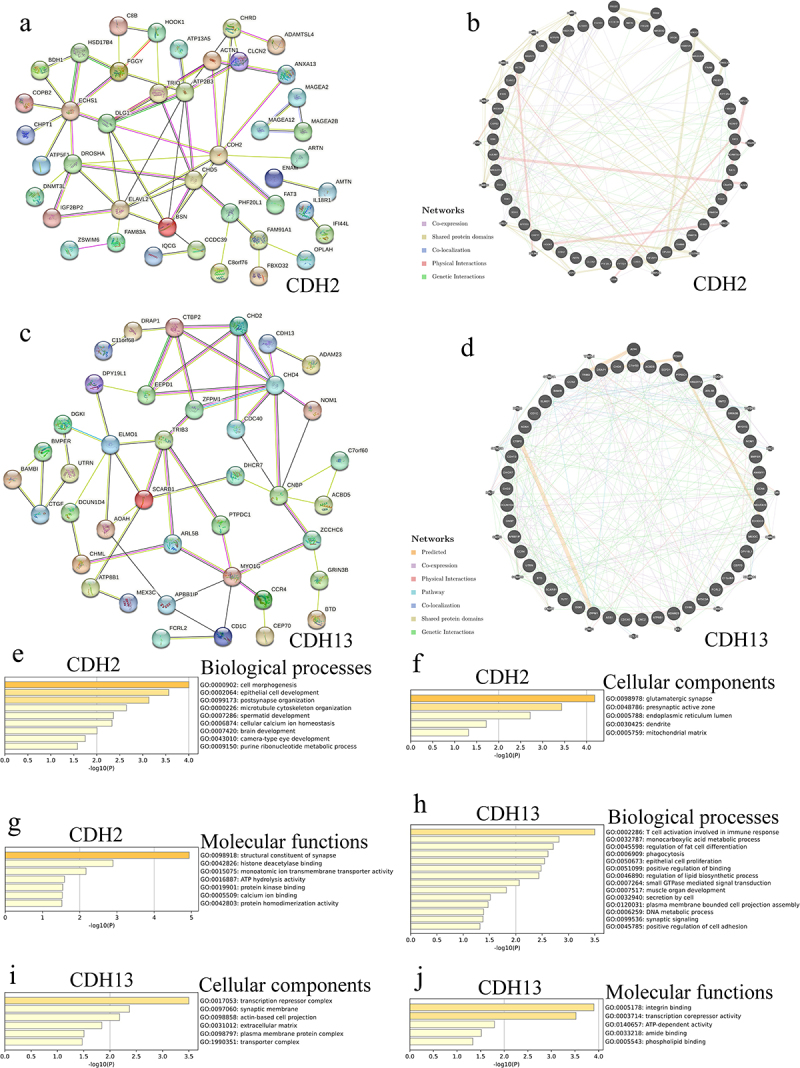
Interaction and function analyses of *CDH2*, *CDH13*, and their altered neighboring genes (ANGs) in adrenocortical carcinoma (ACC). (a) Protein – protein interaction (PPI) network of *CDH2* and its ANGs in patients with ACC (STRING); (b) Network analyses of *CDH2* and its ANGs in patients with ACC (GeneMANIA); (c) PPI network of *CDH13* and its ANGs in patients with ACC (STRING); (d) network analyses of *CDH13* and its ANGs in patients with ACC (GeneMANIA); (e) biological processes of *CDH2* and its ANGs in patients with ACC (Metascape); (f) cellular components of *CDH2* and its ANGs in patients with ACC (Metascape); (g) molecular functions of *CDH2* and its ANGs in patients with ACC (Metascape); (h) biological processes of *CDH13* and its ANGs in patients with ACC (Metascape); (i) cellular components of *CDH13* and its ANGs in patients with ACC (Metascape); (j) Molecular functions of *CDH13* and its ANGs in patients with ACC (Metascape).

We noted *CDH2* and *CDH13* altered neighboring gene (ANG) alteration frequencies of ≥ 33.33% and ≥ 25.00%, respectively, among the 50 most frequent ANGs in patients with ACC (Tables 1 and 2). The most frequent ANGs for *CDH2* in patients with ACC were *PKHD1* (66.67%), *PHF20L1* (50.00%), and *KCNH7* (50.00%) (Table 1). Furthermore, *NT5C3A* (50.00%), *ANKMY1* (50.00%), and *CD1C* (50.00%) were the most frequent ANGs of *CDH13* in patients with ACC (Table 2). We obtained 43 nodes and 124 edges in the protein – protein interaction (PPI) networks of *CDH2* and ANGs (Figure 2a). *CDH2* was predicted to be connected to ANGs by coexpression, shared protein domains, colocalization, physical interactions, and genetic interactions in a complex interaction network (Figure 2b). Moreover, we obtained 40 nodes and 114 edges in the PPI networks of *CDH13* and ANGs (Figure 2c). *CDH13* was predicted to be connected to ANGs by coexpression, physical interactions, pathways, colocalization, shared protein domains, and genetic interactions (Figure 2d).

**Table 1.** The top 50 of *CDH2* neighbor gene alterations in ACC (cBioportal).

Gene	Altered group	Unaltered group	*p*-Value
*DNMT3L*	3 (50.00%)	0 (0.00%)	2.96E-04
*PKHD1*	4 (66.67%)	5 (7.25%)	1.38E-03
*PHF20L1*	3 (50.00%)	0 (0.00%)	2.96E-04
*KCNH7*	3 (50.00%)	1 (1.45%)	1.15E-03
*CHD5*	3 (50.00%)	2 (2.90%)	2.78E-03
*DROSHA*	3 (50.00%)	2 (2.90%)	2.78E-03
*ZSWIM6*	3 (50.00%)	2 (2.90%)	2.78E-03
*ADAMTSL4*	3 (50.00%)	3 (4.35%)	5.38E-03
*ATP2B3*	3 (50.00%)	3 (4.35%)	5.38E-03
*MAGEA12*	3 (50.00%)	3 (4.35%)	5.38E-03
*MAGEA2*	3 (50.00%)	3 (4.35%)	5.38E-03
*MAGEA2B*	3 (50.00%)	3 (4.35%)	5.38E-03
*OPLAH*	3 (50.00%)	3 (4.35%)	5.38E-03
*TRIO*	3 (50.00%)	3 (4.35%)	5.38E-03
*ACTN1*	2 (33.33%)	0 (0.00%)	5.41E-03
*AMTN*	2 (33.33%)	0 (0.00%)	5.41E-03
*ANKRD18DP*	2 (33.33%)	0 (0.00%)	5.41E-03
*ANXA13*	2 (33.33%)	0 (0.00%)	5.41E-03
*ARTN*	2 (33.33%)	0 (0.00%)	5.41E-03
*ATP13A5*	2 (33.33%)	0 (0.00%)	5.41E-03
*ATP5PB*	2 (33.33%)	0 (0.00%)	5.41E-03
*BDH1*	2 (33.33%)	0 (0.00%)	5.41E-03
*BSN*	2 (33.33%)	0 (0.00%)	5.41E-03
*C8B*	2 (33.33%)	0 (0.00%)	5.41E-03
*C8ORF76*	2 (33.33%)	0 (0.00%)	5.41E-03
*CCDC39*	2 (33.33%)	0 (0.00%)	5.41E-03
*CHPT1*	2 (33.33%)	0 (0.00%)	5.41E-03
*CHRD*	2 (33.33%)	0 (0.00%)	5.41E-03
*CLCN2*	2 (33.33%)	0 (0.00%)	5.41E-03
*COPB2*	2 (33.33%)	0 (0.00%)	5.41E-03
*DLG1*	2 (33.33%)	0 (0.00%)	5.41E-03
*DRGX*	2 (33.33%)	0 (0.00%)	5.41E-03
*ECHS1*	2 (33.33%)	0 (0.00%)	5.41E-03
*ELAVL2*	2 (33.33%)	0 (0.00%)	5.41E-03
*ENAM*	2 (33.33%)	0 (0.00%)	5.41E-03
*FAM157A*	2 (33.33%)	0 (0.00%)	5.41E-03
*FAM83A*	2 (33.33%)	0 (0.00%)	5.41E-03
*FAM91A1*	2 (33.33%)	0 (0.00%)	5.41E-03
*FAT3*	2 (33.33%)	0 (0.00%)	5.41E-03
*FBXO32*	2 (33.33%)	0 (0.00%)	5.41E-03
*FGGY*	2 (33.33%)	0 (0.00%)	5.41E-03
*FRG2B*	2 (33.33%)	0 (0.00%)	5.41E-03
*FYTTD1*	2 (33.33%)	0 (0.00%)	5.41E-03
*HJURP*	2 (33.33%)	0 (0.00%)	5.41E-03
*HOOK1*	2 (33.33%)	0 (0.00%)	5.41E-03
*HSD17B4*	2 (33.33%)	0 (0.00%)	5.41E-03
*IFI44L*	2 (33.33%)	0 (0.00%)	5.41E-03
*IGF2BP2*	2 (33.33%)	0 (0.00%)	5.41E-03
*IL18R1*	2 (33.33%)	0 (0.00%)	5.41E-03
*IQCG*	2 (33.33%)	0 (0.00%)	5.41E-03

**Table 2.** The top 50 of *CDH13* neighbor gene alterations in ACC (cBioportal).

Gene	Altered group	Unaltered group	*p*-Value
*NT5C3A*	2 (50.00%)	0 (0.00%)	2.16E-03
*ANKMY1*	2 (50.00%)	1 (1.41%)	6.37E-03
*CD1C*	2 (50.00%)	1 (1.41%)	6.37E-03
*CHD2*	2 (50.00%)	1 (1.41%)	6.37E-03
*CTBP2*	2 (50.00%)	1 (1.41%)	6.37E-03
*FCRL2*	2 (50.00%)	1 (1.41%)	6.37E-03
*TRIB3*	2 (50.00%)	1 (1.41%)	6.37E-03
*ECHDC3*	2 (50.00%)	2 (2.82%)	0.0125
*NDUFA10*	2 (50.00%)	2 (2.82%)	0.0125
*PTPDC1*	2 (50.00%)	2 (2.82%)	0.0125
*SCARB1*	2 (50.00%)	2 (2.82%)	0.0125
*CCN2*	2 (50.00%)	4 (5.63%)	0.0301
*ENTREP3*	2 (50.00%)	4 (5.63%)	0.0301
*FAM241A*	2 (50.00%)	4 (5.63%)	0.0301
*MEX3C*	2 (50.00%)	4 (5.63%)	0.0301
*NOM1*	2 (50.00%)	4 (5.63%)	0.0301
*TUT7*	2 (50.00%)	4 (5.63%)	0.0301
*ZFPM1*	2 (50.00%)	4 (5.63%)	0.0301
*GRIN3B*	2 (50.00%)	5 (7.04%)	0.0413
*MYO1G*	2 (50.00%)	5 (7.04%)	0.0413
*UTRN*	2 (50.00%)	5 (7.04%)	0.0413
*ACBD5*	1 (25.00%)	0 (0.00%)	0.05
*ADAM23*	1 (25.00%)	0 (0.00%)	0.05
*AOAH*	1 (25.00%)	0 (0.00%)	0.05
*APBB1IP*	1 (25.00%)	0 (0.00%)	0.05
*ARL5B*	1 (25.00%)	0 (0.00%)	0.05
*ASB1*	1 (25.00%)	0 (0.00%)	0.05
*ATP8B1*	1 (25.00%)	0 (0.00%)	0.05
*BAMBI*	1 (25.00%)	0 (0.00%)	0.05
*BMPER*	1 (25.00%)	0 (0.00%)	0.05
*BMT2*	1 (25.00%)	0 (0.00%)	0.05
*BTD*	1 (25.00%)	0 (0.00%)	0.05
*C11ORF68*	1 (25.00%)	0 (0.00%)	0.05
*C7ORF33*	1 (25.00%)	0 (0.00%)	0.05
*CCN6*	1 (25.00%)	0 (0.00%)	0.05
*CCR4*	1 (25.00%)	0 (0.00%)	0.05
*CDC40*	1 (25.00%)	0 (0.00%)	0.05
*CEP70*	1 (25.00%)	0 (0.00%)	0.05
*CHD4*	1 (25.00%)	0 (0.00%)	0.05
*CHML*	1 (25.00%)	0 (0.00%)	0.05
*CMC2*	1 (25.00%)	0 (0.00%)	0.05
*CNBP*	1 (25.00%)	0 (0.00%)	0.05
*DCUN1D4*	1 (25.00%)	0 (0.00%)	0.05
*DGKI*	1 (25.00%)	0 (0.00%)	0.05
*DHCR7*	1 (25.00%)	0 (0.00%)	0.05
*DPY19L1*	1 (25.00%)	0 (0.00%)	0.05
*DPY19L2P1*	1 (25.00%)	0 (0.00%)	0.05
*DRAP1*	1 (25.00%)	0 (0.00%)	0.05
*EEPD1*	1 (25.00%)	0 (0.00%)	0.05
*ELMO1*	1 (25.00%)	0 (0.00%)	0.05

### Gene ontology function and Kyoto encyclopedia of genes and genomes pathway enrichment analysis of CDH2, CDH13, and their ANGs in ACC

The biological processes associated with *CDH2* and ANGs in patients with ACC were mainly associated with cell morphogenesis, epithelial cell development, postsynaptic organization, microtubule cytoskeleton organization, spermatid development, cellular calcium ion homeostasis, brain development, camera-type eye development, and purine ribonucleotide metabolism (Figure 2e). Moreover, glutamatergic synapses, presynaptic active zones, endoplasmic reticulum lumen, dendrites, and the mitochondrial matrix were the main cellular components of *CDH2* and its ANGs in patients with ACC (Figure 2f). The molecular functions of *CDH2* and its ANGs in patients with ACC included structural constituents of synapses, histone deacetylase binding, monoatomic ion transmembrane transporter activity, ATP hydrolysis activity, protein kinase binding, calcium ion binding, and protein homodimerization activity (Figure 2g). The biological processes related to *CDH13* and its ANGs in patients with ACC were T-cell activation involved in immune, monocarboxylic acid metabolic process, regulation of fat cell differentiation, phagocytosis, epithelial cell proliferation, positive regulation of binding, regulation of lipid biosynthetic process, small GTPase-mediated signal transduction, muscle organ development, secretion by cell, plasma membrane-bound cell projection assembly, DNA metabolic process, synaptic signaling, and positive regulation of cell adhesion (Figure 2h). Additionally, the main cellular components of *CDH13* and its top 50 ANGs in patients with ACC were the transcription repressor complex, synaptic membrane, actin-based cell projection, extracellular matrix, plasma membrane protein complex, and transporter complex (Figure 2i). The molecular functions of *CDH13* and its ANGs in patients with ACC included integrin binding, transcription corepressor activity, ATP-dependent activity, amide binding, and phospholipid binding (Figure 2j).

### MiRNA and kinase targets of CDH2 and CDH13 in patients with ACC

Using LinkedOmics, we found the miRNA targets of *CDH2* and *CDH13* (Table 3). MiR-331, miR-486, and miR-24 were the targets of *CDH2* in ACC (*p* < .001) (Table 3). The miRNA targets of *CDH13* in ACC were miR-101, miR-142-3P, and miR-527 (*p* < .001) (Table 3). Moreover, we found that DYRK1B, LYN, and NLK were the kinase targets of *CDH2* in patients with ACC (*p* < .001) (Table 4). The kinase targets of *CDH13* were TTK, CDK2, and CHEK1 in patients with ACC (*p* < .001) (Table 4).

**Table 3.** The top three miRNA target of *CDH2* and *CDH13* in ACC (LinkedOmics).

Gene	Gene set	Leading Edge Number	*P*-value
*CDH2*	CCAGGGG,miR-331	27	<2.2e-16
	GTACAGG,miR-486	13	<2.2e-16
	CTGAGCC,miR-24	45	<2.2e-16
*CDH13*	GTACTGT,miR-101	65	<2.2e-16
	ACACTAC,miR-142-3P	50	<2.2e-16
	CTTTGCA,miR-527	57	<2.2e-16

**Table 4.** The top three kinase target of *CDH2* and *CDH13* in ACC (LinkedOmics).

Gene	Kinase target	Description	Leading edge number	*P*-value
CDH2	Kinase_DYRK1B	dual specificity tyrosine phosphorylation regulated kinase 1B	2	<2.2e-16
	Kinase_LYN	LYN proto-oncogene, Src family tyrosine kinase	24	<2.2e-16
	Kinase_NLK	nemo like kinase	5	<2.2e-16
CDH13	Kinase_TTK	TTK protein kinase	9	<2.2e-16
	Kinase_CDK2	cyclin dependent kinase 2	102	<2.2e-16
	Kinase_CHEK1	checkpoint kinase 1	42	<2.2e-16

### Correlation of differentially expressed genes and CDH2 and CDH13 expression in patients with ACC

**Figure 3.**
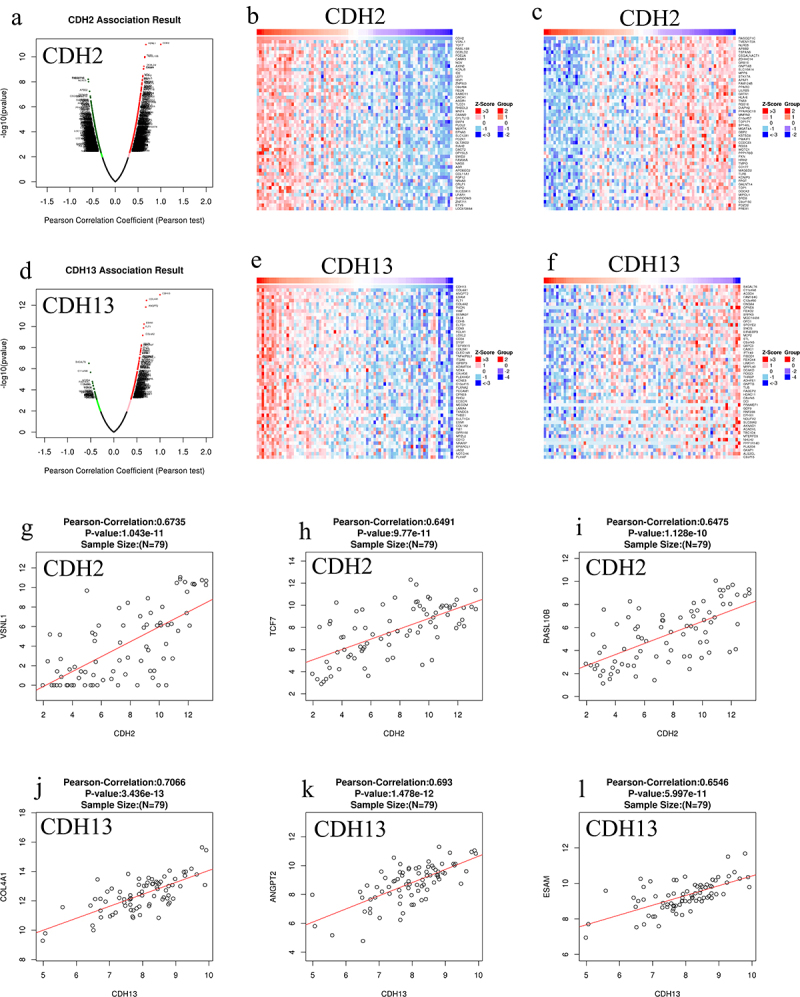
Genes differentially expressed in correlation with *CDH2* and *CDH13* expression in adrenocortical carcinoma (ACC) (obtained using LinkedOmics). (a and d) the Pearson test was used to analyze correlations between *CDH2*, *CDH13*, and genes differentially expressed in ACC, respectively; (b, c, e, and f) heat maps showing genes positively and negatively correlated with *CDH2* and *CDH13* in ACC, respectively (top 50 genes); the scatter plot shows Pearson correlation of *CDH2* and *CDH13* expression with expression of *VSNL1* (g), *TCF7* (h), *RASL10B* (i), *COL4A1* (j), *ANGPT2* (k), and *ESAM* (l) in ACC; red and blue indicate positively and negatively correlated genes, respectively.

A total of 4,824 and 2,748 genes were found to be closely related to *CDH2* and *CDH13*, respectively, in patients with ACC (Figure 3a–d). Among them, 2,096 and 1,898 genes showed positive correlation and 2,728 and 850 genes showed negative correlation with *CDH2* and *CDH13* expression, respectively (Figure 3a–d). Fifty genes showed significant positive and negative correlation with *CDH2* and *CDH13* expression in patients with ACC (Figure 3b,c,e,f). The expression of *CDH2* was strongly and positively associated with *VSNL1* (Pearson correlation coefficient [PCC] = 0.6735, *p* = 1.043e-11; Figure 3g), *TCF7* (PCC = 0.6491, *p* = 9.77e-11; Figure 3h), and *RASL10B* (PCC = 0.6475, *p* = 1.128e-10; Figure 3i). The expression of *CDH13* was positively correlated with *COL4A1* (PCC = 0.7066, *p* = 3.436e-13; Figure 3j), *ANGPT2* (PCC = 0.693, *p* = 1.478e-12; Figure 3k), and *ESAM* (PCC = 0.6546, *p* = 5.997e-11; Figure 3l) expression.

### Correlation of immune cell infiltration and CDH13 expression and anti-PD1/CTLA-4/PD-L1 immunotherapy in ACC

**Figure 4.**
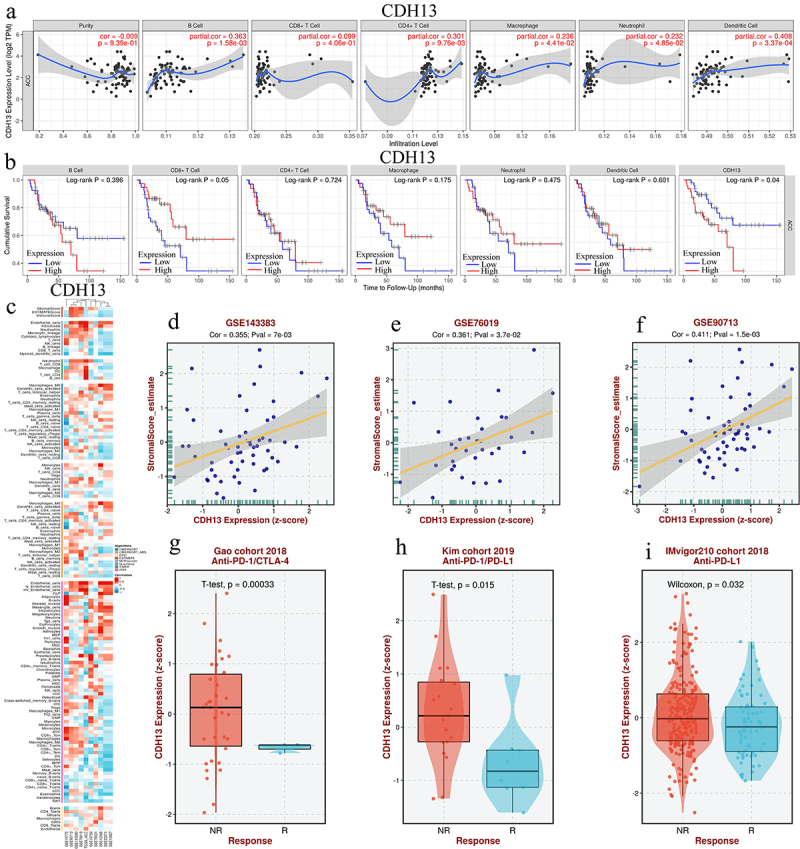
The correlation between *CDH13* expression and immune cell infiltration and anti-PD1/CTLA-4/PD-L1 immunotherapy in adrenocortical carcinoma (ACC). (a) The correlation between *CDH13* expression and immune cell infiltration levels in patients with ACC (TIMER); (b) the cumulative survival curve of *CDH13* and immune cell infiltration in patients with ACC (TIMER); (c) heat maps showing the correlation between *CDH13* and immune cell infiltration in ACC (BEST); (d-f) the correlation between *CDH13* expression and immune score in patients with ACC (BEST); (g and i) boxplot showing the correlation between *CDH13* expression and anti-PD1/CTLA-4/PD-L1 immunotherapy in ACC (BEST).

The expression levels of *CDH13* in patients with ACC were positively associated with immune cell infiltration (B cells, CD4+ T cells, macrophages, neutrophils, and dendritic cells) (*p* < .05; Figure 4a–f). The cumulative survival of patients with ACC was longer than that of patients with low CD8+ T-cell expression levels (*p* = .05; Figure 4b). However, the cumulative survival of patients with ACC was longer in those with low *CDH13* expression levels *(p* = .04; Figure 4b). Moreover, *CDH13* expression in patients with ACC treated with anti-PD1/CTLA-4, anti-PD1PD-L1, and anti-PD-L1 was significantly downregulated (*p* = .05) (Figure 4g–i).

### Therapeutic drugs of CDH2 and CDH13 in ACC

**Figure 5.**
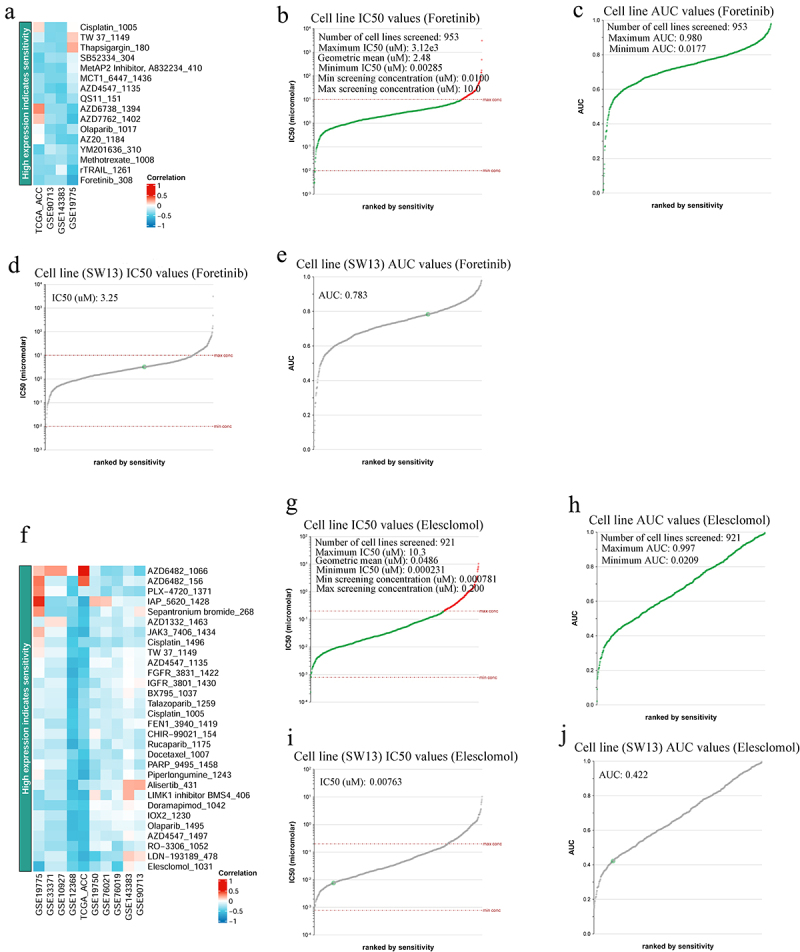
IC_50_ evaluation of foretinib and elesclomol in different tissue types of cancer. (a and f) heat maps showing *CDH2* and *CDH13* low expression indicates resistance drugs ranking, respectively (BEST); (b) IC_50_ values of foretinib for the different cell lines (genomics of drug sensitivity in Cancer); (c) area under the curve (AUC) values of foretinib for the different cell lines (genomics of drug sensitivity in Cancer); (d) IC_50_ values of foretinib for the SW13 cell line (genomics of drug sensitivity in Cancer); (e) AUC values of foretinib for the SW13cell line (genomics of drug sensitivity in Cancer); (g) IC_50_ values of elesclomol for the different cell lines (genomics of drug sensitivity in Cancer); (h) AUC values of elesclomol for the different cell lines (genomics of drug sensitivity in Cancer); (i) IC_50_ values of elesclomol for the SW13 cell line (genomics of drug sensitivity in Cancer); (j) AUC values of elesclomol for the SW13 cell line (genomics of drug sensitivity in cancer).

Using the BEST database, we predicted foretinib and elesclomol as the top drug candidates for targeting CDH2 and CDH13, respectively (Figure 5a–f). Next, the genomics of drug sensitivity in the cancer database was used to evaluate the inhibitory effects of foretinib and elesclomol on an ACC cell line (SW13). Foretinib inhibited 953 cell lines with area under the curve (AUC) values greater than 0.980 (Figure 5c) and had a good inhibitory effect on these cell lines (0.00285 ≤ IC_50_ [μM] ≤ 3120) (Figure 5b). Furthermore, foretinib had a strong inhibitory effect on SW13 (an ACC cell line) (AUC = 0.783, IC_50_ [μM] = 3.25) (Figure 5d,e). However, elesclomol inhibited 921 cell lines, with AUC values greater than 0.0209 (Figure 5h) and had a good inhibitory effect on these cell lines (0.000231 ≤ IC_50_ [μM] ≤ 10.3) (Figure 5g). Elesclomol had a strong inhibitory effect on SW13 cells (AUC = 0.422, IC_50_ [μM] = 0.00763) (Figure 5i,j).

## Discussion

Abnormal expression of *CDH13* has been reported in various tumors. However, its expression in patients with ACC remains unknown. CDH13 expression is often downregulated in cancer cells. Low CDH13 expression is associated with poor prognosis in various cancers, such as lung, ovarian, cervical, and prostate cancer.^13^ Notably, we found that the expression of *CDH13* was strongly upregulated in patients with ACC, and low expression was related to a good prognosis in ACC patients. However, *CDH2* expression was strongly downregulated in patients with ACC. Downregulation of N-cadherin has been reported in ACC.^14^ *CDH2* expression levels were lower in male patients than in female patients with ACC; patients exhibiting low *CDH2* expression had longer survival times than those with high expression. The number of female patients with ACC generally exceeds that of male patients (1.5:1).^15,16^ Whether a sex-related difference in *CDH2* expression is an important factor affecting the prognosis of patients with ACC warrants further investigation. The transcript levels of *CDH2* and *CDH13* in patients with ACC aged >65 years were significantly lower than those in patients aged <65 years. Does this indicate an age advantage in the survival rate of patients with ACC? In this context, it is pertinent to mention that the survival rate of children with ACC who underwent surgery was lower than that of adults with ACC.^17^ Next, we attempted to explain the abnormalities in *CDH2* and *CDH13* expression through genetic alterations in patients. The expression of *CDH2* and *CDH13* was altered in 9% and 15% of patients with ACC, respectively. Abnormal expression of *CDH2* and *CDH13* caused by genetic changes may also be an important factor. DNA methylation affects the abnormal expression of *CDH2* and *CDH13* in cancer patients.^18,19^ However, this hypothesis warrants further investigation. These results suggest that *CDH2* and *CDH13* may serve as potential therapeutic and prognostic markers in patients with ACC.

*CDH2*, *CDH13*, and their ANGs are linked to a complex interaction network through coexpression and physical and genetic interactions. The molecular functions of *CDH2* and its ANGs mainly include histone deacetylase binding, monoatomic ion transmembrane transporter activity, ATP hydrolysis, protein kinase binding, and calcium ion binding. This shows that *CDH2* and its ANGs may affect gene expression, cell cycle, and energy metabolism by regulating histone acetylation, protease activity, and ion channels, ultimately affecting tumor proliferation, differentiation, and metastasis. The molecular functions of *CDH13* and its ANGs in patients with ACC include integrin binding, transcription corepressor activity, ATP-dependent activity, amide binding, and phospholipid binding. Thus, *CDH13* and its ANGs may regulate the proliferation, invasion, migration, and angiogenesis of cancer cells by affecting integrin function, gene transcription, ability metabolism, and amide and phospholipid metabolism. Taken together, the functions involving *CDH2*, *CDH13*, and their ANGs may be involved in the occurrence and progression of ACC. Therefore, the regulation of these genes may be a potential treatment strategy for ACC.

Mining miRNA and kinase targets of key genes is an important breakthrough in ACC treatment. We found that miR-331, miR-486, miR-24, miR-101, miR-142-3P, and miR-527 are targets of *CDH2* and *CDH13* in patients with ACC. MiR-331, miR-24, miR-101, and miR-527 are associated with tumor cell proliferation, migration, invasion, and drug resistance, and may, therefore, be promising targets for cancer therapy.^20–22^ However, their relationship with ACC has not yet been reported. Furthermore, miR-486-3p may inhibit ACC cell proliferation by reducing the production of fatty acid synthases and fatty acids.^23^ Our results also indicate that miR-101, miR-142-3P, and miR-527 are targets of *CDH13* in patients with ACC. In a previous study, we showed that miR-142-3P might be an important regulatory target in ACC.^24^ We investigated the kinase targets of *CDH2* and *CDH13* in patients with ACC. We found that DYRK1B, LYN, NLK, TTK, CDK2, and CHEK1 were the kinase targets of *CDH2* and *CDH13*. DYRK1B is a serine/threonine kinase involved in tumor progression and cell proliferation. Silencing or inactivation of DYRK1B may be a potential therapeutic strategy in cancer.^25^ Overexpression of LYN promotes the proliferation, migration, and invasion of cervical cancer cells by activating the IL-6/STAT3 pathway. Thus, it could be used as a novel target for the treatment of cervical cancer.^26^ NLK is a key regulator in many cancers. Lentivirus-mediated *NLK* knockout inhibited the growth and metastasis of small cell lung cancer; therefore it can be used as a potential target for the treatment of small cell lung cancer.^27^ However, its role in ACC has not yet been clarified. Furthermore, high expression of TTK, CDK2, and CHEK1 has been reported in ACC, which may play an important role in ACC progression and serve as potential biomarkers for future diagnosis and treatment.^28–30^ In summary, these miRNAs and kinases may serve as potential therapeutic targets for ACC.

We explored the correlation between the differentially expressed genes and *CDH2* and *CDH13* expression in patients with ACC. The expression of 4,824 and 2,748 genes was correlated with *CDH2* and *CDH13* expression, respectively. Among these, *VSNL1*, *TCF7*, *RASL10B*, *COL4A1*, *ANGPT2*, and *ESAM* were the top six genes whose expression was positively correlated with the expression of *CDH2* and *CDH13*. Therefore, targeting these genes may provide additional therapeutic options for ACC. Immune infiltration is closely associated with tumor progression and prognosis.^31^ Cancer immunotherapy has led to significant advances in the treatment of multiple cancers. As expected, the expression levels of *CDH13* in patients with ACC were positively correlated with immune cell infiltration. Targeting *CDH13* or its related regulatory targets may be a feasible strategy for improving the immune microenvironment in patients with ACC. We also found that high levels of CD8+ T-cell infiltration can possibly prolong the survival of patients with ACC. However, our results show that *CDH13* expression is not related to the infiltration of CD8+ T cells. *CDH13* and its ANGs can also activate T cells. These findings provide new avenues for ACC immunotherapy using CD8+ T cells. Furthermore, we found that the expression of *CDH13* in patients with ACC who were administered anti-PD1/CTLA-4/PD-L1 was strongly downregulated. Thus, patients with ACC who are treated with anti-PD1/CTLA-4/PD-L1 antibodies may have a better prognosis. However, the role of immunotherapy in ACC is limited.^32^ Studies have shown that the RTK signaling pathway inhibitor, foretinib, and the HSP90 inhibitor, elesclomol, have good antitumor effects and are safe.^33,34^ However, many tyrosine kinase inhibitors against ACC (sunitinib, cabozantinib, and linsitinib) have been evaluated and have failed to obtain good results,^35^ but the effect of foretinib on ACC remains unclear. We evaluated the inhibitory effects of foretinib and elesclomol on SW13 (while SW13 may no longer be considered the ACC model, it was the only one analyzed owing to a lack of available information on H295R in the database). Foretinib and elesclomol exhibited broad-spectrum inhibitory effects on cancer cell lines. Foretinib and elesclomol may exert strong inhibitory effects on SW13 cells by inhibiting the expression of *CDH2* and *CDH13*. Therefore, these drugs may be effective for the treatment of ACC. We identified the roles of *CDH2* and *CDH13* in ACC using bioinformatics methods. However, further validation through in vitro and ex vivo experiments is necessary to confirm their relationship.

In summary, our results provide insights into the expression, gene regulatory network, prognostic value, therapeutic targets, and drugs against *CDH2* and *CDH13* in patients with ACC. Our findings provide a better understanding of the pathogenesis of ACC and could aid in devising effective treatment strategies. *CDH2* and *CDH13* may be potential prognostic and therapeutic targets of ACC.

## Materials and methods

### GEPIA

We used GEPIA (http://gepia.cancer-pku.cn/index.html) to analyze the relationships between gene expression, tumor pathological stages, and prognosis. The screening criteria were as follows: (1) genes: *CDH2* and *CDH13*; (2) dataset: ACC; and (3) 77 patients; threshold-setting conditions: *P*-value cutoff = 0.05. The Student’s *t*-test was used to analyze the expression of *CDH2* and *CDH13* in ACC. Kaplan – Meier curves were used to analyze the prognosis of patients with ACC.^24^

### UALCAN

UALCAN (http://ualcan.path.uab.edu/analysis.html) is a comprehensive, user-friendly, and interactive web resource for mining and analysis of cancer data, mainly from The Cancer Genome Atlas (TCGA) database. We used UALCAN to analyze the expression of *CDH2* and *CDH13* in ACC. The “Expression Analysis” module of the UALCAN database was used to analyze TCGA gene expression data; the screening criteria were set as follows: (1) genes: *CDH2* and *CDH13*; (2) dataset: ACC; (3) 79 ACC patients (31 male and 48 female); threshold setting conditions: *P*-value cutoff = 0.05. The Student’s *t*-test was used for comparative analysis.^24^

### BEST

BEST (https://rookieutopia.com/app_direct/BEST/) provides a curated database and innovative analytical pipelines to explore cancer biomarkers at a high resolution. Protein expression, immune cell infiltration, candidate agents, and immunotherapy targeting *CDH2* and *CDH13* in ACC were analyzed using BEST. The “Clinical association,” “Cell infiltration,” “Immunotherapy,” and “Candidate agents” modules of the BEST database were used to analyze gene expression omnibus and TCGA gene expression data using the following screening criteria: (1) genes: *CDH2* and *CDH13*; (2) dataset: ACC (10 datasets and 508 patients).^24^

### cBioPortal

cBioPortal (http://cbioportal.org) is an online database used for tumor gene mutation analysis. We used cBioPortal to analyze alterations in *CDH2*, *CDH13*, and the top 50 ANGs. A total of 75 ACC samples were analyzed, and z-scores for mRNA expression relative to all samples (log RNA Seq V2 RSEM) were obtained using a z-score threshold of ± 2.0.^24^

### STRING and GeneMANIA

STRING (https://string-db.org/cgi/input.pl.) and GeneMANIA (http://www.genemania.org.) are online databases used for analyzing gene – protein and PPI networks. STRING was used to build a low-confidence level (0.150) PPI network and screen criteria for species defined as humans. GeneMANIA was used to explore the functions of *CDH2*, *CDH13*, and their top 50 ANGs.^24^

### Metascape

Metascape (https://metascape.org) is an online database used to analyze the functions and signaling pathways of genes and proteins. We used Metascape to analyze the functions and signaling pathways of *CDH2*, *CDH13*, and their top 50 ANGs.^24^

### LinkedOmics

LinkedOmics (http://www.linkedomics.org/) is a public online platform for analyzing correlations between differentially expressed genes related to tumor target genes and for predicting miRNA and kinase targets. It was used to identify kinase targets, miRNA targets, and differentially expressed genes related to *CDH2* and *CDH13*.^24^

### TIMER

TIMER (https://cistome.shinyapps.io/timer/) is an online database used to analyze the relationship between tumor genes and infiltrating immune cells. We used it to analyze the correlation between *CDH2* and *CDH13* expression and immune cell infiltration.

### Genomics of drug sensitivity in cancer analysis

Genomics of drug sensitivity in cancer (http://www.cancerRxgene.org) is a specialized public database for obtaining information on potential anticancer drugs. We used this database to identify drugs targeting *CDH2* and *CDH13* and to predict their anti-ACC activity.^24^

## Data Availability

The datasets used and/or analyzed during the current study are available from the corresponding author on reasonable request.
